# Understanding the effects of reductions in local government expenditure on food safety services in England, 2009–10 to 2019–20: a longitudinal ecological study

**DOI:** 10.1136/bmjopen-2025-107146

**Published:** 2026-01-09

**Authors:** Lauren Murrell, Helen E Clough, Xingna Zhang, Roger Gibb, Marie Anne Chattaway, Mark A Green, Iain Edward Buchan, Ben Barr, Daniel Hungerford

**Affiliations:** 1 National Institute for Health and Care Research Health Protection Research Unit in Gastrointestinal Infections, University of Liverpool, Liverpool, UK, Liverpool, UK; 2 Clinical Infection, Microbiology & Immunology, University of Liverpool Institute of Infection Veterinary and Ecological Sciences, Liverpool, UK; 3 Department of Livestock and One Health, Institute of Infection, Veterinary and Ecological Sciences, University of Liverpool, Liverpool, UK; 4 Department of Public Health and Policy, University of Liverpool Faculty of Health and Life Sciences, Liverpool, UK; 5 Health Protection Research Unit Gastrointestinal Infection, University of Liverpool, Liverpool, UK, Public Contributor, PPI Advisor to, Liverpool, UK; 6 Gastrointestinal Bacteria Refence Unit, UKHSA, London, England, UK; 7 NIHR Health Protection Research Unit in Genomics and Enabling Data, University of Warwick, Warwick, UK, Warwick, UK; 8 Geography & Planning, University of Liverpool, Liverpool, UK; 9 Department of Public Health, Policy and Systems, University of Liverpool, Liverpool, Merseyside, UK; 10 Public Health and Policy, University of Liverpool, Liverpool, UK; 11 Clinical Infection, Microbiology and Immunology, University of Liverpool Institute of Infection Veterinary and Ecological Sciences, Liverpool, UK; 12 University of Liverpool, Health protection research unit gastrointestinal ifnections University of Liverpool Liverpool, UK L69 3GF, Liverpool, UK

**Keywords:** Public health, Epidemiology, Gastrointestinal infections

## Abstract

**Abstract:**

**Objective:**

To understand how reductions in resource allocation affect food safety services in England.

**Design:**

This longitudinal ecological study analysed secondary observational data.

**Setting:**

England, data at the local authority level.

**Participants:**

Ecological study, without human participants.

**Primary and secondary outcome measures:**

The primary outcome measures were the number of staff, represented by the number of full-time equivalents per capita, number of interventions per establishment, and proportion of hygiene-compliant establishments.

**Results:**

A £1 decrease in food safety expenditure per capita was associated with a 2% (−3.3 to –0.7) decrease in staffing levels and a 1.6% (–3.2 to –0.1) decrease in the number of interventions achieved per establishment. A one-unit reduction in staff was associated with a 42.2% (–80.5 to –11.9) decrease in the number of interventions achieved. No evidence of an association was found between expenditure or staff levels and the proportion of compliant establishments.

**Conclusions:**

Spending reductions negatively affected the capacity of food safety teams to provide key services. Reductions in food safety expenditure significantly affected food hygiene staff levels and service provision. This finding raises concerns about the capacity of food safety teams to operate and the potential for increased public risk of gastrointestinal infections.

**STRENGTHS AND LIMITATIONS OF THIS STUDY**

- This study links routinely collected data from diverse sources to provide valuable public health insights.
- This study uses longitudinal ecological design with national coverage, allowing for the assessment of changes in food safety service staffing and activity levels across all local authorities in England.
- The use of routinely collected administrative data sets provides consistent measures of food safety service indicators over time and enables the assessment of how changes in exposure relate to changes in service indicators.
- As an ecological study, the analysis cannot make individual-level inferences; therefore, estimates reflect area level associations rather than causal relationships at the individual level.
- Further research is needed to understand the effect of local authority cuts and food safety service provision on gastrointestinal disease outcomes.

## Introduction

###  Background

Approximately 17 million cases of gastrointestinal infections are reported annually in the UK,^1^ making them a significant public health concern. These illnesses often result in diarrhoea and vomiting and, in some cases, death.^2^ Gastrointestinal infections are usually spread through contaminated food, water and surfaces.^3^ Approximately 2.4 million cases of foodborne illness^4^ and 16 400 hospitalisations^2^ are estimated per year in the UK, with around 180 deaths occurring annually.^5^ Additionally, the total societal cost associated with foodborne diseases is approximately £9.1 billion per year, including lost earnings, disturbance to business, medical costs and costs associated with missed school.^4^

In England, local authorities (LAs) are responsible for enforcing food safety^6^ and preventing foodborne illness and outbreak. Food safety is provided by environmental and regulatory (ER) services and is a statutory service, which means that LAs must provide it. The Food Standards Agency (FSA) is the central authority that oversees food safety controls enforced by LAs.^6^ These controls are delivered by environmental health (EH) staff,^7^ also called EH officers or EH practitioners (EHPs). Food safety services include food hygiene inspections to ensure food is safe from bacterial contamination,^7^ testing food samples, food safety advice and investigation of food poisoning outbreaks and foodborne illness.^8^ ER services under food safety are likely important for the prevention of gastrointestinal infections.

The financial crisis of 2008 saw austerity measures implemented by central government to control national debt. This resulted in funding cuts to LA services across England with reductions in spending of up to 50%.^9^ Although ER services are largely statutory, there have been substantial cuts to these services, which disproportionately affected poorer areas. Per capita spending on food safety and infection control services declined by 22.8% in the most deprived areas compared with 6.3% in the least deprived areas between 2009−10 and 2020−21.^10^ Food establishments in more deprived areas are 25% less likely to be food hygiene compliant^11^; therefore, reducing food safety resources may exacerbate the existing inequalities in food hygiene compliance.

Certain signs reveal that local funding cuts are negatively affecting LA services. However, many services lack data on outputs or outcomes, which makes evaluating the impact of expenditure reductions challenging.^12^ Since 2010, LA services have seen staff reductions, such as in food safety services.^7 13^ There has also been a reduction in waste collection, library service points, bus services,^12^ reports of food standards, health and safety focusing on high-risk businesses and scaling back proactive work that may prevent serious risks.^14^

The extent to which local funding cuts have affected food safety services is unreported. Thus, this study aimed to evaluate the impact of reductions in food safety expenditure on food hygiene staff, number of interventions achieved and proportion of broadly compliant establishments.

## Methods

A longitudinal study at an LA level was conducted using financial data from 2009−10 to 2019−20. The study focused on England and used data from 314 lower tier LAs that are responsible for environmental services in England. In total, 26 LAs were removed from the final analysis. Thirteen LAs with missing food safety expenditure data were excluded. Five were also excluded because food hygiene returns were reported with food standards, and another six were removed because of issues with consistency in LA reporting. LAs in the Isles of Scilly and City of London were excluded due to low population sizes and distinct funding structures. The LA geographical boundaries used were the 2020 boundaries. In instances where LA data were not at this level, data were aggregated and transformed to the 2020 level to provide consistent data for this period.

### Local authority data

#### Expenditure data

Expenditure data were sourced from the Place-based Longitudinal Data Resource,^15^ which collates publicly available annual LA outturn data for cultural, environmental and regulatory services reported to the UK central government’s department of housing and local government.^16^ Annual food safety expenditure, by financial year, was defined as spending reported in budget outturns for environmental and regulatory services under the title of food safety. This was adjusted for inflation using gross domestic product deflator^17^ and then normalised by Office for National Statistics (ONS) mid-year population estimates to provide food safety services expenditure per capita.

#### Service indicator data

We defined three LA food safety service indicators. First, the annual number of interventions carried out per establishment. Interventions include inspections, sampling, audits, monitoring, surveillance and intelligence gathering, as well as advice and education.^18^ LAs use a risk-based approach to inform intervention frequency and type (table 1). Data were obtained from the FSA’s LA enforcement monitoring system (LAEMS)^19^ and through a freedom of information request where data were not immediately available. More information is provided in section 1 of the online supplemental material.

**Table 1:** Further background information on data

Food hygiene interventions include inspections, sampling, audits, monitoring, surveillance and intelligence gathering as well as advice and education. Interventions are carried out by environmental health staff.			
The type and frequency of interventions are determined by the risk the establishment faces to the public	Risk rating	Frequency and intervention type	
	A	</6 months	Inspection, partial inspection or audit
	B	</12 months	Inspection, partial inspection or audit
	C	</18 months	Inspection, partial inspection or audit (if broadly compliant interventions can alternate between the above and another type of official control)
	D	</24 months	Alternate between inspection, partial inspection or audit and other types of intervention
	E	Every 3 years or an alternative programme	Alternative enforcement strategy

The food hygiene rating score ranges from 0 to 5, which reflects the standards of hygiene on the day of inspection. Only establishments that directly supply the public will receive a food hygiene rating score. The food hygiene score is determined after inspection, audit or partial audit.

Table 1 uses information adapted from the Food Law Code of Practice, Annex 1 of Food Law Code of Practice and the Food Standards Agency website.^23 38^

Second, food hygiene full-time-equivalent (FTE) position figures were sourced from LAEMS, as a measure of staff per capita, normalised using population estimates from the ONS.^20^ The food hygiene FTE refers to professional or administrative staff who are allocated to food hygiene food law enforcement work.^21^ This measure indicates resource level. The term staff or staffing levels will be used when referring to FTE (see section 1.3 of the online supplemental material for more details).

Third, the proportion of broadly compliant food establishments (cafes, restaurants, pubs and food shops)^22^ was defined as the number of businesses that received a rating of ≥3, divided by the number of establishments rated that year. Food hygiene scores range from 0 to 5, reflecting the food hygiene standards found on the day of inspection.^22^ A score of 0 means urgent improvement is required, and 5 means that the hygiene standards are very good ^23^ (see section 1.4 of the online supplemental material for more details). These data were obtained from the Consumer Data Research Centre, which sources and compiles data from the FSA.^23^ Data were aggregated from the business level to the LA level and transformed to financial year for consistent reporting across all data. The food hygiene rating scheme (FHRS) data were analysed for the period from 2012−13 to 2019−20 because the implementation of the FHRS scheme in 2010 likely reduced data for 2010–2012. Given the role of LA staff in food safety and its enforcement, changes in staff levels or interventions may influence hygiene standards. Therefore, we use broad compliance for food hygiene as an indicator of establishment performance to allow analysis of how changes to trends in staffing levels, number of interventions and financial allocation may influence businesses’ hygiene compliance.

### Missing data

Food safety expenditure data were missing, with 58 LAs not reporting expenditure data for at least 1 year. This could be due to reporting under another spending line or LAs not disaggregating at the required level. This may have also contributed to missing data in outcome variables of interest, such as staff and interventions. In some instances, LAs reported food standards returns only or food standards combined with food hygiene returns, and such LAs were removed. We attributed LAs missing data for FHRS to the roll-out of the scheme in late 2010, as there were no missing data after 2012. LAs with >50% data missing were removed, and multiple imputation was used to estimate the values for remaining missing data (see section 2 of online supplemental material for more information).

### Analysis

Data were analysed descriptively by year, deprivation level, LA type and rural or urban classification (see section 3 in online supplemental material). Fixed-effects panel regression models were then used to estimate the change in the number of FTE per capita, number of interventions achieved per establishment and proportion of compliant establishments associated with each £1 decrease in food safety expenditure per capita. All models included a fixed effect for each LA to remove all between LA differences, so the models only estimate the association between exposure and outcomes within LAs over time. All models also use robust clustered standard errors; this accounts for clustering within LAs. All models included year as a continuous variable, allowing us to control for temporal variation.

First, the number of FTEs was modelled as the outcome, with food safety per capita as the exposure. A log-linear regression model with the log population estimate as an offset was used to model FTE per capita. The model was weighted by the LA population size. Second, the number of interventions per establishment was analysed as the outcome with food safety expenditure as the exposure. A log-linear regression model with the number of establishments as the offset was used to model the number of interventions per establishment. In addition, the model was weighted by the number of establishments in each LA. Then, the number of interventions per establishment was modelled as the outcome with food safety per capita and FTE per 10 000 as the exposures. A log-linear regression model with the log of the number of establishments as the offset was used. This model was weighted by the number of establishments.

Binomial regression with logit link function was employed to model the effect of food safety expenditure per capita on the proportion of broadly compliant establishments, specifying the outcomes as a grouped binomial proportion, necessitating the inclusion of the number of compliant out of the total establishments rated as a weight.^24^ This model used the number of FTE per 10 000 as the main exposure, with the proportion of broadly compliant establishments as the outcome. Finally, FTE per 10 000 of the population and food safety expenditure per capita were modelled as the exposures on the proportion of compliant establishments.

### Patient and public involvement

A patient and public involvement and engagement (PPIE) panel was held at the beginning of the research project where input from panel members contributed to initial planning. Following this, a PPIE member was a contributor to the current study, contributing to the study design and conceptualisation, dissemination of this work and co-authored this paper.

## Results

Table 2 shows the summary statistics of our measures of interest. The mean food safety expenditure per capita decreased from £3.14 in 2009–10 to £2.27 per capita by 2019−20. Over the same period, the average number of FTEs per 10 000 population in England reduced from 0.31 to 0.24 per 10 000 population. The average number of interventions achieved decreased by 13 246 per 100 000 establishments, and the average percent of broadly compliant establishments increased from 88% to 94%.

**Table 2:** Summary table of measures of interest

Year	Average food safety expenditure per capita (£)	Average number of full-time equivalent staff positions per 10 000 population	Average number of interventions achieved per 100 000 establishments	Average percent of broadly compliant establishments		
				Average percent of broadly compliant establishments	Average number of broadly compliant establishments	Average number of establishments rated in the FHRS
2009−10	3.14	0.31	73 497			
2010−11	3.20	0.30	71 628	–		
2011−12	2.85	0.29	69 660	–		
2012−13	2.79	0.28	68 210	88%	518	600
2013−14	2.66	0.27	66 500	89%	538	611
2014−15	2.45	0.27	64 315	90%	522	590
2015−16	2.44	0.26	64 513	92%	528	582
2016−17	2.34	0.25	62 773	92%	546	603
2017−18	2.32	0.25	62 887	93%	570	625
2018−19	2.30	0.25	62 657	93%	586	637
2019−20	2.27	0.24	60 251	94%	582	625

FHRS, food hygiene rating scheme.

**Figure 1:**
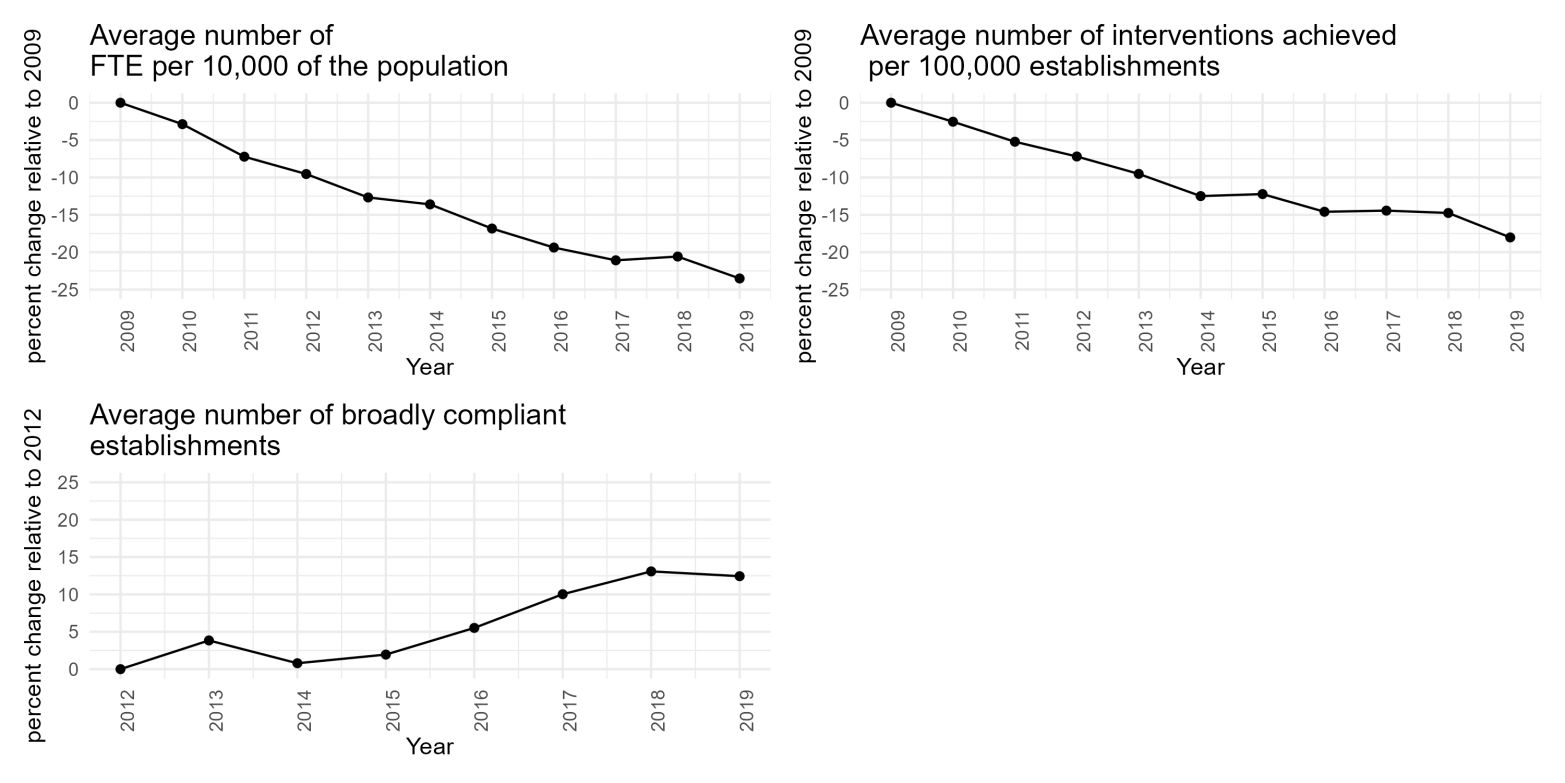
Percentage change in the outcomes of interest relative to baseline year 2009 for measures of FTE, interventions and proportion of compliant establishments. The year represents the financial year. FTE, full-time equivalent

Figure 1 presents the percentage change in outcomes of interest relative to 2009−10. The number of FTE per 10 000 reduced by 23.5% in 2019−20. The number of interventions achieved per 100 000 establishments reduced by 18% in 2019. From 2012 to 2018, the average number of broadly compliant establishments increased by 13% before dropping to 12% in 2019/20 compared with 2009−10. More details on the exposures and outcomes of interest by deprivation, LA structure and rural or urban categorisation are provided in section 3 of the online supplemental material.

Table 3 presents the results from the regression analyses. The number of staff per capita decreased by 2.0% (p<0.01), with each £1 per capita reduction in food safety expenditure. The number of interventions per establishment saw a significant decrease of 1.6% (p<0.05) with a £1 per capita decrease in food safety expenditure. For each FTE per 10 000 population decrease in staff, the number of interventions per establishment decreased by 42.2% (p<0.01). When accounting for the number of FTE, a £1 reduction in expenditure was not significantly associated with the number of interventions, which decreased by 1.4% (p>0.05). When accounting for spend per capita, a reduction in staff was significantly associated with a decrease of 39.4% (p<0.01) in the number of interventions achieved per establishment. No significant relationship was found between food safety expenditure or FTE and the odds of establishments achieving broadly compliant ratings. We carried out a sensitivity analysis on data with missing values present, and the results are provided in section 4 of the online supplemental material.

**Table 3:** Regression analysis results showing the effect of food safety expenditure and staffing levels on primary outcomes

Outcome	Exposure: food safety expenditure per capita (£1 decrease)	Exposure: number FTE per 10 000 population (one-unit decrease)	Exposure: food safety expenditure per capita (£1 decrease) + number FTE per 10 000 population (one-unit decrease)
Percent change in the number of FTE per capita	−2% (−3.3 to –0.7)**	–	
Percent change in the number of interventions achieved per establishment	−1.6% (−3.2 to –0.1)*	−42.2% (−80.5 to –11.9)**	Expenditure: −1.4% (−2.9 to 0.1) FTE: −39.4% (−76.1 to –10.3)**
Percent change in the odds of being rated broadly compliant	−0.9% (−4.2 to 2.3)	13.4 (−34.2 to 44.2)	Expenditure: −1% (−4.3 to 2.2) FTE: 15% (−32.1 to 45.3)

Each exposure variable appears as a column heading, and each outcome appears as a row heading. Each cell reports the result of a separate regression model. Sample sizes (N) and R2 for linear models are included in online supplemental material table 5.1.

'***' 0.001 '**' 0.01 '*' 0.05 '.' 0.1 ' ' 1.

FTE, full-time equivalent.

## Discussion

This study shows for the first time how the reduction in local funding cuts is significantly associated with the capacity of local statutory food safety services. Lower food safety expenditure was associated with a decrease in food hygiene staff and the number of interventions achieved. We find no significant relationship in relation to hygiene compliance.

Our findings are consistent with reports of staff cuts across public services in recent years due to austerity. Staff reduction is a key mechanism by which authorities try to make savings.^13^ This is reflected in a National Audit Office (NAO) report, which revealed that food hygiene staff decreased by 13% between 2012−13 and 2017−18,^7^ highlighting that even statutory services are vulnerable to cuts during austerity. This has been an approach taken by most authorities, with 96% of single-tier authorities and 86% of district councils reducing the number of staff between 2011 and 2013.^25^ Some LAs are reducing higher pay-grade staff, including managers.^26^ The loss of senior staff with more experience may create a workforce with less knowledge and expertise, a noted concern of some council workers.^27^ A survey by the Chartered Institute of Environmental Health described that 56% of LA’s reported vacancies in EH teams remain unfilled for at least 6 months, primarily due to the lack of qualified or experienced EHPs.^28^ No official UK guidance has been established on the benchmark number of staff per portion of the population or per number of establishments, and it may be argued that the previous figures on staff cuts could indicate an initial surplus in staff. However, concerns raised from the Chartered Institute of Environmental Health and reporting from the NAO regarding the continuous staff cuts and the potential impact on food safety enforcement suggest this is unlikely to be the case.^7 29 30^ Staff cuts and reduced ability to hire experienced staff into EH roles may create a workforce that lacks depth and overall capacity to handle the rising demand in of services.

Although important actions such as food hygiene inspections have been prioritised with spending cuts,^31^ our results show that a decrease in staff has affected the number of interventions achieved. These reductions in staff may also help explain the association between expenditure and interventions achieved. The reduction in staff means that the workforce is becoming more stretched with fewer staff having larger workloads^31 32^ and with professionally qualified food hygiene staff carrying out more inspections and audits per person in 2019−20 than in 2010−11.^9^ In addition, LAs failing to meet intervention targets noted staffing shortfalls as the reason and expressed concerns regarding the impact on food safety.^7^ They also noted changes in functions due to staff cuts, for example, cutting back on activities such as advice, guidance and sampling, prioritising higher risk premises and outsourcing to third-party contractors to increase inspection capacity.^7^ This is in line with our results and suggests a decrease in service capacity, which may increase the risk of a foodborne disease outbreak.

Evidence shows that the capacity of EH teams is key for public protection, with workforce experience, workforce levels and regulation compliance having protective effects against foodborne illness.^33^ Literature shows that interventions such as training food handlers in food safety and hygiene are effective in reducing microbial prevalence in food service premises.^34^ Reducing access to advice and education visits, or other interventions, may cause a decline in the practice of food handlers and increase the occurrence of microbial contamination, placing the public at greater risk of gastrointestinal infection. A previous work highlighted the importance of EHP inspection visits, with 87.8% (n=387) of survey respondents in one study saying that the action taken following the inspection had improved the hygiene of their establishment.^35^ As a substantial amount of foodborne illness outbreaks are associated with settings that rely on intervention and guidance from EHPs, our results are troubling. This is particularly true when considering the increasing trend of hospitalisations due to pathogens linked to foodborne disease.^36 37^

Although our results highlight challenges surrounding the capacity of food safety teams and delivery of service, we saw no association between food safety expenditure or staffing levels and food hygiene compliance. Paradoxically, we see an increase in broadly compliant establishments during the study period, agreeing with other reports during this time.^7^ However, these results must be interpreted with caution. Although our results indicate that a reduction in resources does not affect hygiene compliance rates, other factors may be involved, such as the requirement of LAs to achieve compliance as stated in the food law code of practice.^38^ Staff prioritise establishments of higher risk,^31^ which means that businesses achieving lower FHRS scores will likely be a resource priority to achieve compliance. The FHRS score offers a snapshot of the hygiene practice at the time of inspection. Although important, it provides limited detail concerning the hygiene practice of the establishments between inspections. There may be gaps in compliance by area or deprivation level, reflective of funding inequalities, exemplified in our analysis and previous research.^10^

Technology, advanced diagnostic tools as an assistive resource to EH workers and businesses may play a role in combatting the effects of resource reduction and helping maintain standards. The FSA reported the implementation of artificial intelligence (AI) throughout the food system stages of supply, production, processing, distribution, consumption and waste; however, more research into the current use of AI in the UK food system is needed.^39^ In addition to technology, establishments can employ private consultant companies to help reach and maintain compliance. Overall compliant food hygiene scores may show a level of resilience of food safety teams and food business operators to resource cuts; however, this does not eliminate the risk of outbreaks.

The reduction in expenditure and consequent trends occur when demands are increasing, as the number of registered food establishments is increasing.^31^ Inequalities in funding cuts to these services may widen health inequalities,^10^ with recent studies showing reduced capacity for gastrointestinal infection work in the more deprived areas and lower rates of food hygiene compliance.^11 40^ Evidence supports that investment in food and sanitation services is associated with a reduction in the incidence of diseases caused by key food- and water-related pathogens.^41^ These service cuts may place the public at greater risk to these infections. Each £1 per capita reduction in food safety expenditure is associated with 2% of full-time staff lost and over 40% decrease in the number of interventions achieved per establishment. When put into context, these numbers may have a real impact. In practice, this could look like longer waiting times, reduced capacity across roles, workers limited to reactive work and potential risk to public safety. Although we do not investigate the effect on gastrointestinal infections, our next line of research sets out to detail the association with changes in expenditure and service capacity on gastrointestinal infection outcomes.

### Strengths and limitations

Unlike previous studies, this study attempts to identify the extent to which reduced expenditure may affect services and their operation and capacity. We use data on key indicators of food safety in the context of food hygiene functions to track trends. This study has several limitations. First, we rely on ecological data; however, this work allows us to explore important general trends of local funding cuts on these services across England. Second, this study focuses primarily on food safety services. Although these services are important, gastrointestinal infections can spread through other routes, including person-to-person transmission within communities, transmission from contact with animals or exposure while travelling outside of England. Third, there were limitations to data quality, such as missing data, which was mitigated by using multiple imputation. Additionally, reduced expenditure and staffing could have affected data collection and quality, such as with compliance, potentially introducing bias. Funding cuts may have altered data collection and may, in part, reflect altered operational practices in time of fiscal constraint. Finally, directly relating expenditure decrease to FTE outcomes may oversimplify results, as overhead allocation and fixed departmental costs were not accounted for.

## Conclusion

In this study, we quantify the impact of food safety expenditure reductions on indicators of food hygiene functions, crucial for prevention of foodborne infectious disease. Although ER and food safety and infection control services have seen changes in service expenditure over time, there is little to no knowledge of the association between local funding cuts and impact on service delivery. The results show that decreases in food safety expenditure significantly affected staffing levels and service provision. Although this raises concern about the capacity of food safety teams to protect the public, we find no effect on hygiene compliance. This work raises questions about the effect of these trends on public risk to gastrointestinal infection and any inequalities that may be present.

## Supplementary material

10.1136/bmjopen-2025-107146 online supplemental file 1

## Data Availability

Most data used are publicly available, some data used are now subject to restrictions, or were accessed by a freedom of information request.
