# Supplementary material for: Understanding the effects of reductions in local government expenditure on food safety services in England, 2009–10 to 2019–20: a longitudinal ecological study
Source: BMJ Open. 2026 Jan 9;16(1):e107146. doi: 10.1136/bmjopen-2025-107146 (PMC12815172; doi:10.1136/bmjopen-2025-107146)
Supplement: Supplementary file 2 [file bmjopen-2025-107146.reviewer_comments.pdf]

PEER REVIEW HISTORY

BMJ Open publishes all reviews undertaken for accepted manuscripts. Reviewers are asked to complete a checklist review form and are provided with free text boxes to elaborate on their assessment. These free text comments are reproduced below.

This paper was submitted to another journal from BMJ but declined for publication following peer review. The authors addressed the reviewers' comments and submitted the revised paper to BMJ Open. The paper was subsequently accepted for publication at BMJ Open.

ARTICLE DETAILS

Title (Provisional)

Understanding the impacts of reductions in local government expenditure on food safety services, England 2009/10 - 2019/20, a longitudinal ecological study

Authors

Murrell, Lauren; Clough, Helen E; Zhang, Xingna; Gibb , Roger; Chattaway, Marie Anne; Green, Mark A; Buchan, Iain Edward; Barr, Ben; Hungerford, Daniel

---

VERSION 1 - REVIEW

**Reviewer**	**1**
**Name**	**Reviewer 1**
**Affiliation**	
**Date**	**21-May-2025**
**COI**	**no competing interest**

Comments not published pending reviewer's consent

**Reviewer**	**2**
**Name**	**Reviewer 2**
**Affiliation**	
**Date**	**21-May-2025**
**COI**	**no competing interest**

Comments not published pending reviewer's consent

VERSION 2 - REVIEW

**Reviewer**	**3**
**Name**	**Hoffmann, Vivian**
**Affiliation**	**International Food Research & Policy Institute (IFPRI)**
**Date**	**11-Nov-2025**
**COI**	**None**

This is a nicely done paper. I have only minor comments:

1. I am confused by the use of a binomial regression model to estimate a model in which the outcome is the proportion of compliant establishments in a local authority (page 6, lines 3-4). Or is the analysis done at the establishment level with a binary outcome? This should be clarified in the text.

2. It would also be helpful to present the regression results shown in Table 3 with additional details (number of observations in each model, R-squared value for linear models), make clear that each of the estimates shown comes from a separate regression model, and indicate which variables are outcomes vs. exposures.

Very minor / grammatical issues

Page 3: spell out ONS

Page 5, lines 25 and 32: “food safety per capita” – should this be food safety expenditure as the outcome?

page 9, line 8: we find no significant relationship was found in relation to hygiene compliance

page 10, line 35: Such as…. (sentence fragment)

VERSION 2 - AUTHOR RESPONSE

Reviewer: 3
Dr. Vivian Hoffmann, International Food Research & Policy Institute (IFPRI)
Comments to the Author:
This is a nicely done paper. I have only minor comments:

1. I am confused by the use of a binomial regression model to estimate a model in which the outcome is the proportion of compliant establishments in a local authority (page 6, lines 3-4). Or is the analysis done at the establishment level with a binary outcome? This should be clarified in the text.

Thank you for raising this point. The analysis is not at establishment level, although underlying data were at establishment level, for the purpose of this analysis data were

aggregated to local authority year- level. The outcome is therefore the proportion of establishments that were broadly compliant in each local authority each year. Because we observe the number of broadly compliant (numerator) and the total number of establishments rated (denominator), we model this as a grouped binomial outcome supplying the proportion and denominator to the model. We have also added in a supporting reference.

I have now updated the language to reflect the approach and make the methodology used clearer, please see page 6 line 34-37

"We used binomial regression with logit link function to model the effect of food safety expenditure per capita on the proportion of broadly compliant establishments, specifying the outcomes as a grouped binomial proportion, necessitating the inclusion of the number compliant out of the total establishments rated as a weight (24)."

2. It would also be helpful to present the regression results shown in Table 3 with additional details (number of observations in each model, R-squared value for linear models), make clear that each of the estimates shown comes from a separate regression model, and indicate which variables are outcomes vs. exposures.

Thank you for this suggestion, I have now clearly indicated the exposures and outcomes in the table. I also have added a note directly beneath the table to make it clear that each estimate is derived from a separate regression model. In addition, I have included a note to direct the reader to the R-squared values and number of observations, which are in the supplementary material page 10, table S51. These details were placed in the supplementary material to avoid overcrowding the main results table and to maintain clarity.

"*Each exposure variable appears as a column heading, and each outcome appears as a row heading. Each cell reports the result of a separate regression model. Sample sizes (N) and R^2 for linear models are included in supplementary material Table S5.1." page 8 lines 16-18."*

Very minor / grammatical issues

Page 3: spell out ONS

Thank you this has now been corrected and reads as

"Secondly, we sourced food hygiene full-time-equivalent (FTE) position figures from LAEMS, as a measure of staff per capita, normalized using population estimates from the Office for National statistics (ONS)." Page 4, lines 23-24.

Page 5, lines 25 and 32: "food safety per capita" – should this be food safety expenditure as the outcome?

Thank you for your questions. Here we model food safety expenditure per capita as the exposure, to allow us to understand how a change in expenditure might impact the number of staff in an area, in addition to the number of interventions they achieve. I have made this clearer by indicating in Table 3 what measures are outcome and what are exposure.

page 9, line 8: we find no significant relationship was found in relation to hygiene compliance

Thank you, this detail will be added to read, "We find no significant relationship in relation to hygiene compliance" on page 9 line 5.

page 10, line 35: Such as…. (sentence fragment)

Thank you for highlighting this, I have now edited to read "Secondly, this paper focuses primarily on food safety services. Although these services are important, GI infections can spread through other routes, including person-to-person transmission within communities, transmission from contact with animals, or exposure whilst travelling outside of England." Page 11 line 44, page 12 line 1-2.
